# Norovirus Transmission on Cruise Ship

**DOI:** 10.3201/eid1101.040434

**Published:** 2005-01

**Authors:** Elmira T. Isakbaeva, Marc-Alain Widdowson, R. Suzanne Beard, Sandra N. Bulens, James Mullins, Stephan S. Monroe, Joseph Bresee, Patricia Sassano, Elaine H. Cramer, Roger I. Glass

**Affiliations:** * Centers for Disease Control and Prevention, Atlanta, Georgia, USA; † Atlanta Research and Education Foundation, Atlanta, Georgia, USA; ‡ Bucks County Department of Health, Doylestown, Pennsylvania, USA

**Keywords:** viral gastroenteritis, outbreak, Norwalk, cruise ship, dispatch

## Abstract

We describe an investigation of a norovirus gastroenteritis outbreak aboard a cruise ship affecting 6 consecutive cruises and the use of sequence analysis to determine modes of virus transmission. Noroviruses (NoV), are the most common cause of infectious acute gastroenteritis and are transmitted feco-orally through food and water, directly from person to person and by environmental contamination (*1*). These viruses are often responsible for protracted outbreaks in closed settings, such as cruise ships, nursing homes, and hospitals (*2**,**3*).

## Abstract 2

We documented transmission by food and person-to-person contact; persistence of virus despite sanitization onboard, including introductions of new strains; and seeding of an outbreak on land.

On November 20, 2002, cruise ship X recorded an elevated number of persons with acute gastroenteritis symptoms reporting to the ship’s infirmary (84 [4%] of 2,318 passengers) during a 7-day vacation cruise from Florida to the Caribbean. According to federal regulations, when the incidence of acute gastroenteritis among passengers and crew exceeds 3%, an outbreak is defined and requires a formal investigation (*4*). The outbreak continued on the subsequent cruise (cruise 2), after which the vessel was removed from service for 1 week of aggressive sanitization. Despite cleaning, gastroenteritis also developed in 192 (8%) of 2,456 passengers and 23 (2.3%) of 999 crew on the following cruise (cruise 3). To determine the source of this continuing outbreak and to better understand the mechanisms of NoV transmission, we began an investigation on cruise 1 and collected stool specimens from persons with gastroenteritis on this cruise and the next 5 cruises.

## The Study

We surveyed all 2,318 passengers on cruise 1 to determine dates of illness onset, symptoms, cabin locations, activities, and food consumption. We also performed a sanitary inspection of the ship. We suspected that initial infection among passengers on cruise 1 originated from a common food or water source and then continued to spread from person to person. Therefore, we conducted a case-control study with all passengers in whom illness developed early in the cruise (days 3 and 4) after embarkation (defined as day 1) and also with passengers who became ill later (day 5). Controls were systematically selected among passengers who reported no symptoms of gastroenteritis throughout the entire cruise. We continued to monitor the number of cases of acute gastroenteritis on the subsequent 5 cruises and collected fecal specimens from ill persons on all 6 cruises. During our shipboard investigation, we also obtained stool specimens from ill persons in a long-term care facility affected by an outbreak of acute gastroenteritis, in which the index patient was a passenger who returned ill to the facility after disembarking from cruise 1. All stool specimens were tested for NoV by reverse transcription–polymerase chain reaction, as previously described (*5*). The positive amplicons were sequenced, and sequences were compared for genetic diversity.

**Figure 1:**
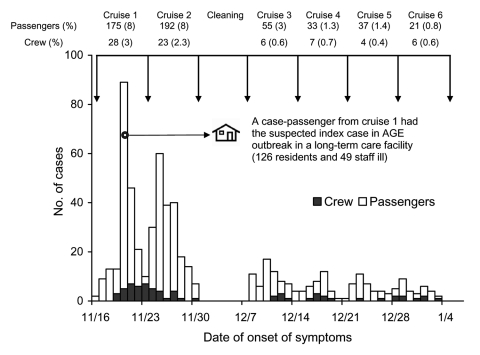
Number (%) of cases of acute gastroenteritis among 513 passengers and 74 crew by date of symptom onset reported to the infirmary on 6 consecutive cruises of ship X, November 2002–January 2003. Arrows indicate start and end of each cruise. pax; passengers.

The outbreak began abruptly on day 2 of cruise 1 and continued on cruise 2 with new passengers. Despite sanitization of the ship for 1 week after cruise 2, illness was also reported among passengers on cruise 3 (Figure 1). On the subsequent cruises (*4*–*6*), the number of ill persons reporting to the infirmary remained above background levels but below 3%. Of the 2,318 surveyed passengers on cruise 1, 1,276 (55%) returned questionnaires, of these, 212 case-passengers and 265 control-passengers were enrolled in our study. We identified that eating breakfast at restaurant A on day 2 of the cruise was associated with illness among case-passengers with onset of symptoms on day 3 (odds ratio [OR] 4.04, p < 0.01) and that eating dinner at the same restaurant on day 2 was a risk factor for illness among case-passengers who became ill on day 4 (OR 2.8, p < 0.005). We also found that eating dinner at restaurant B aboard ship on day 3 was associated with illness among case-passengers with onset on day 5 (OR 2.3, p < 0.05). Restaurants A and B did not share a galley. Case-passengers with later onset of illness on day 5 were more likely than controls to have a cabin mate in whom gastroenteritis developed on days 3 or 4 (OR 2.01, p = 0.01), which suggests either infection by environmental contamination or by person-to-person spread.

**Figure 2:**
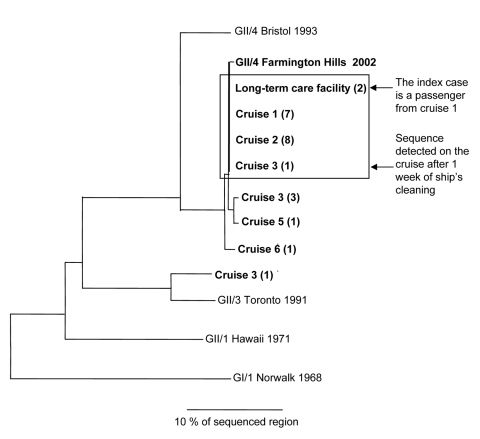
Phylogram of 9 norovirus sequence types detected in outbreaks on ship X, 4 reference sequences from GenBank, and the Farmington Hills virus. The tree is based on a 277-nucleotide region (region C) of the capsid gene and was created using uncorrected distances calculated by the DISTANCES program (Genetics Computer Group, Madison, WI) and was constructed by neighbor-joining using the GROWTREE program version 10.3 (Genetics Computer Group). Numbers in parenthesis indicate number of samples with the identical sequence detected in a given outbreak. Box highlights sequence types belonging to the Farmington Hills virus that are indistinguishable in region C. GenBank accession no. for reference strains include Bristol virus, X76716; Toronto virus, U02030; Hawaii virus, U07611; and Norwalk virus, M87661. Scale bar represents 10% divergence.

Of 55 tested stool specimens from all 6 cruises, 25 (45%) were positive for NoV and belonged to 6 strains (Table). Norovirus was detected in at least 1 stool sample from all cruises, except cruise 4, where no stool samples were found positive, and in 2 samples of ill persons from the long-term care facility (Table). The genetic sequences detected on cruises 1 and 2 were identical in regions B and C and belonged to a lineage of NoV within genogroup II (GII), cluster 4 (Figure 2), which has been provisionally described as the Farmington Hills strain (*6*). Five of the 8 NoV-positive specimens on cruise 3, which sailed after sanitization, contained 3 different sequences (X, Y, and Z). Sequence X was found in 1 sample and was identical to the sequence detected on cruises 1 and 2, which suggested that this strain may have persisted onboard despite cleaning. Sequence Y was found in 3 samples and differed from sequence X by 3 nucleotides (nt) in region C, which suggested that it was the predominant strain and probably newly introduced by passengers or crew at the start of cruise 3. Sequence Z was detected in 1 sample and belonged to the same lineage of NoV as the strain found on cruises 1 and 2 but to a different cluster (cluster 3), which suggested that it was also newly introduced onto cruise 3. Single stool samples from persons on cruises 5 and 6 contained a sequence that differed from the Farmington Hills strain by 3 nt and 1 nt, respectively, which suggested probable continuous reintroductions of closely related viruses aboard the ship. A sequence indistinguishable from that found on cruises 1 and 2 was also detected in stool samples from 2 persons ill in the outbreak that occurred in the long-term care facility, which suggested that virus was possibly introduced by the ill passenger from cruise 1. The environmental inspection of the ship identified no major violations.

**Table:** Results of laboratory testing for norovirus in stool specimens, November 2002–January 2003*

Outbreak identification	No. samples			No. sequences detected
	Tested	Positive (%)	Sequenced	
Cruise 1	12	7 (58)	7	1
Cruise 2	14	8 (57)	8	1
Cleaning	1	0 (0)	0	0
Cruise 3	15	8 (53)	5	3
Cruise 4	6	0 (0)	0	0
Cruise 5	3	1 (33)	1	1
Cruise 6	4	1 (25)	1	1
Long-term care facility	2	2 (100)	2	1

*Specimens were collected from ill persons in linked outbreaks of acute gastroenteritis on a cruise ship and in a long-term care facility.

## Conclusions

We report on a large outbreak of NoV-related gastroenteritis that affected 6 consecutive cruises on 1 ship and recurred despite thorough sanitization after the second cruise. In the past, investigations of shipboard outbreaks of viral gastroenteritis were limited by the lack of adequate molecular methods for detecting and characterizing viruses (*7*). In this investigation, epidemiologic analysis suggested an initial foodborne source of infection with subsequent secondary spread from person to person, while molecular analysis provided several new insights into disease transmission. Application of genetic sequencing documented persistence of the same strain onboard between cruises by detecting identical sequences in stool samples from ill passengers before and after 1 week of the vessel’s cleaning. Although these findings suggest that environmental contamination may have helped perpetuate the outbreak, infected crew members could have also been a reservoir of infection between cruises. Molecularly fingerprinting detected viruses confirmed several introductions of new strains aboard, which underscores the difficulty in controlling outbreaks of NoV on cruise ships. Sequence analysis provided evidence that an outbreak of NoV in the care facility was caused by a person returning ill from an outbreak-affected cruise.

Like other outbreaks of viral gastroenteritis on cruise ships (*3*,*6*,*8*–*11*), this outbreak affected several hundred people, was transmitted by multiple modes, and recurred on subsequent cruises. Multiple routes of NoV transmission have been documented in other reports, such as that of an outbreak of gastroenteritis among football players, in which initial foodborne transmission of virus and secondary person-to-person spread was demonstrated (*12*). Outbreaks of gastroenteritis aboard cruise ships are similar to those in other closed and crowded settings where identifying and interrupting multiple routes of transmission has proved particularly challenging (*2*,*13*,*14*).

A limitation of this study was that the investigation on cruise 1 started 7 days after the first cases were reported, and because of logistic constraints, surveying was restricted to passengers in a short period before their disembarkation. Thus, we were unable to investigate risk factors for illness among crew and determine if any of the food-handlers were ill. In addition, poor recall of exposures resulted in a lack of complete data for a detailed evaluation of risk factors. We also did not perform a full investigation on the subsequent cruises because the number of ill persons did not exceed 3%.

Our investigation suggests that efforts to control gastroenteritis outbreaks on cruise ships should address all possible modes of NoV transmission, including foodborne, environmental persistence, and person-to-person spread. Such measures should include extensive disinfection, good food and water handling practices, isolating ill persons, providing paid sick leave for ill crew, and promoting hand-washing with soap and water among passengers and crew. Developing strategies and incentives to dissuade symptomatic passengers from boarding may also minimize opportunities to introduce new strains aboard. Cruise ship outbreaks with <3% of passengers reporting ill should be considered for investigation because they may contribute substantial information on the transmission and epidemiologic characteristics of NoV, which could be used to develop control strategies and prevent future outbreaks on land and at sea.
